# Medial meniscus extrusion is invariably observed and consistent with tibial osteophyte width in elderly populations: The Bunkyo Health Study

**DOI:** 10.1038/s41598-023-49868-7

**Published:** 2023-12-20

**Authors:** Yoshifumi Negishi, Haruka Kaneko, Takako Aoki, Lizu Liu, Arepati Adili, Hitoshi Arita, Shinnosuke Hada, Masahiro Momoeda, Hui Huang, Jun Tomura, Suguru Wakana, Jun Shiozawa, Mitsuaki Kubota, Yuki Someya, Yoshifumi Tamura, Shigeki Aoki, Hirotaka Watada, Ryuzo Kawamori, Takako Negishi-Koga, Yasunori Okada, Muneaki Ishijima

**Affiliations:** 1 Department of Medicine for Orthopedics and Motor Organ, Juntendo University Graduate School of Medicine, 2-1-1, Hongo, Bunkyo-ku, Tokyo, 113-8421 Japan; 2 Department of Pathophysiology for Locomotive Diseases, Juntendo University Graduate School of Medicine, Tokyo, Japan; 3 Sportology Center, Juntendo University Graduate School of Medicine, Tokyo, Japan; 4 Department of Community Medicine and Research for Bone and Joint Diseases, Juntendo University Graduate School of Medicine, Tokyo, Japan; 5 Department of Metabolism and Endocrinology, Juntendo University Graduate School of Medicine, Tokyo, Japan; 6 Department of Radiology, Juntendo University Graduate School of Medicine, Tokyo, Japan

**Keywords:** Osteoarthritis, Osteoarthritis

## Abstract

We reported that the full-length width of medial tibial osteophytes comprising cartilage and bone parts correlates with medial meniscus extrusion (MME) in early-stage knee osteoarthritis (OA). However, no data exist on the prevalence of MME and its relationship with osteophytes in the elderly population. 1191 elderly individuals (females 57%; 72.9 years old on average) in the Bunkyo Health Study underwent standing plain radiograph and proton density-weighted MRI on knee joints. MRI-detected OA changes were evaluated according to the Whole-Organ Magnetic Resonance Imaging Score. A new method of assessing the cartilage and bone parts of osteophytes was developed using pseudo-coloring images of proton density-weighted fat-suppressed MRI. Most subjects showed Kellgren-Lawrence grade 1 or 2 radiographic medial knee OA (88.1%), MME (98.7%, 3.90 ± 2.01 mm), and medial tibial osteophytes (99.3%, 3.27 ± 1.50 mm). Regarding OA changes, MME was closely associated with the full-length width of medial tibial osteophytes (β = 1.114; 95% CI 1.069–1.159; *p* < 0.001) in line with osteophyte width (intraclass correlation coefficient, 0.804; 95% CI 0.783–0.823). Our data revealed that MME and medial tibial osteophytes are observed in the elderly and demonstrate that the degree of MME is consistent with the full-length width of medial tibial osteophytes, suggesting that osteophytes might be implicated in MME.

## Introduction

In a super-aging society, prevention of motor function deterioration is considered to contribute to the extension of healthy life expectancy. Knee osteoarthritis (OA) is a disease in which the motor function decreases with age^1,2^. Nearly 30% of individuals older than 45 years old and over 80% of women older than 80 years old are reported to have radiographic knee OA in the United States and Japan, respectively^3,4^. With the increasing number of elderly people, the number of patients with knee OA is increasing worldwide^5^. Elucidation of the progression mechanism and development of treatment methods for knee OA will thus be important for extending the healthy life expectancy^6,7^.

OA has been defined pathologically as an intrinsic degenerative disease of articular cartilage in which biochemical and metabolic alterations result in its breakdown^8^ and has long been considered a “wear and tear” disease leading to a loss of cartilage^9^. However, accumulated data from molecular biology and experimental studies have demonstrated that many inflammatory mediators produced by cartilage, subchondral bone, and synovium play a pivotal role in the initiation and perpetuation of OA^9^. Analyses of knee OA by focusing on OA features and progressors of early-stage knee OA patients are one of the most important topics in this field^10,11^. After the application of magnetic resonance imaging (MRI) for the diagnosis of knee OA, growing evidence acquired by MRI analyses has indicated that, besides cartilage destruction, alterations in the meniscus, subchondral bone, osteophyte, and synovium are commonly observed in early-stage knee OA^12^, and the changes proceed asymptomatically many years before the onset of knee OA^13,14^. Among these, disorders in the meniscus are considered an important progressive factor for early-stage knee OA^15–17^. Meniscal dysfunction preceded by medial meniscus extrusion (MME) is a risk factor for knee OA^18–20^. More than half of the middle-aged patients with early-stage knee OA have been reported to have MME^21^. Although one of the main causes of MME is meniscus tear^22^, many patients with early-stage knee OA^21^ or established knee OA (10–70% of patients)^23–27^ exhibit MME without meniscus tear. Therefore, an important question that remains to be addressed is how MME develops in early-stage knee OA.

Osteophytes are the most common abnormality among patients with early-stage knee OA who have no radiographic OA^12^. Synovial and/or periosteal mesenchymal stem cells present near the margin of articular cartilage form osteophytes following a process of endochondral ossification^28–31^, thus they are histologically composed of bone tissue capped by cartilage tissue^28^. In our previous study, we reported that both bone and cartilage parts of osteophytes can be readily detected by T2 mapping MRI in the medial tibia in almost all patients with early-stage knee OA^21^ and demonstrated that MME is consistent with the full-length width of medial tibial osteophytes (width of cartilage and bone parts)^21^. In addition, we recently found that anterior meniscus extrusion in elderly subjects is closely associated with the full-length width of the anterior tibial osteophyte^32^. Based on these data, we hypothesized that MME in early-stage knee OA without meniscal tears may be induced through medial displacement of the medial meniscus by the medial tibial osteophyte^21^, since the medial meniscus is tightly attached to the medial tibial plateau by the meniscotibial ligament^21,33,34^. However, our previous study on knee OA was performed in a limited number (n = 50) of patients with early-stage knee OA and joint pain. Little or no information is available on the prevalence of MME and medial tibial osteophytes or their relationship in elderly individuals.

In the present study, elderly individuals in a population-based large cohort, the Bunkyo Health Study (BHS)^32,35–38^, were subjected to radiographic and MRI examinations to analyze knee OA changes. We developed a new method to analyze both the cartilage and bone parts of osteophytes by pseudo-coloring the MRI views and studied the correlations between MME and medial tibial osteophyte width along with the prevalence of MME.

## Results

### Characteristics of the subjects

Among 1630 subjects with BHS, 1191 who underwent both radiography in the standing position and MRI of the knee joint were enrolled in the present study (Table 1). While 43.1% (513 of 1191 subjects) of the subjects were male, 56.9% (678 of 1191 subjects) were female. The mean body mass index (BMI) was 22.8 kg/m^2^ on average. All subjects showed knee joint changes greater than Kellgren-Laurence (K/L) grade 1, and most of the subjects (72.8%, 867 of 1191 subjects) had knee OA with K/L grade 2 on radiography. The femorotibial angle (FTA) was 177.3° on an average. The pain visual analog score (0–100) of the subjects was 8.7 ± 16.3, indicating that the subjects had almost no or weak joint pain.

**Table 1:** Characteristics of the subjects.

N	1191
Age (y) (SD, range)	72.9 (5.4, 65–85)
Gender (male/female)	513 / 678
BMI (kg/m^2^) (SD, Range)	22.8 (3.0, 15.4–36.6)
Radiographic OA severity	K/L1: 177 (14.9%) K/L2: 867 (72.8%) K/L3: 99 (8.3%) K/L4: 48 (4.0%)
FTA (°) (SD, range)	177.3 (2.73, 168–191)

*BMI* body mass index, *K/L* Kellgren–Lawrence grad, *FTA* femoro-tibial angle.

### Application of the pseudo-colored proton density-weighted fat-suppressed (PPDFS) MRI method to osteophyte evaluations

To examine whether or not the PPDFS MRI method could be applied for the evaluation of osteophytes, we compared the findings of osteophytes obtained by proton density-weighted fat-suppressed (PDFS)　MRI, PPDFS MRI, T2 mapping MRI, and histology of surgically removed samples from 10 knee OA patients who underwent uni-compartmental arthroplasty (UKA) (Supplemental Table 1). As shown in Supplemental Fig. 1, the cartilage and bone parts of the medial tibial osteophyte could be more clearly detected by the PPDFS MRI view than by the PDFS MRI view. The cartilage-part width and full-length (cartilage and bone parts) width of osteophytes in each patient were separately measured by T2 mapping MRI, PPDFS MRI, PDFS MRI, and histology, the latter of which was performed on the removed osteophytes (Supplemental Table 2). When the osteophyte widths were compared between histology and PPDFS MRI data, histology and T2 mapping MRI data, or PPDFS MRI and T2 mapping MRI data, the interclass correlation coefficient (ICC) was extremely high, with a mean value of > 0.88. In contrast, the values obtained by comparison between histology and PFDS MRI data, PDFS MRI and PPDFS MRI, or PDFS MRI and T2 mapping MRI data were relatively low (ICC: 0.53 to 0.87) (Supplemental Table 3). Based on these results, the PPDFS MRI method was considered suitable for measuring osteophyte width in subjects in this cohort.

**Figure 1:**
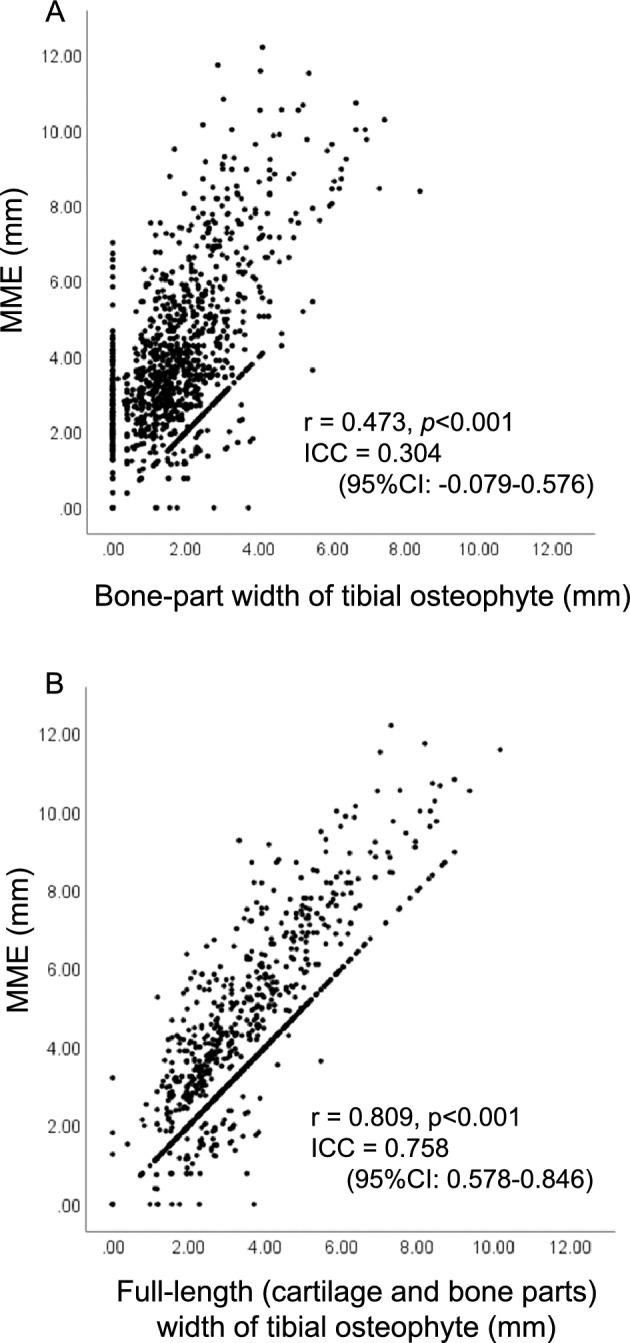
Correlations between medial meniscus extrusion (MME) and medial tibial osteophyte width. Associations between MME and the bone-part width of osteophyte (**A**) and between MME and the full-length (cartilage and bone parts) width of osteophyte (**B**) were analyzed by the Spearman’s rank correlation analysis. r, correlation coefficient; ICC, intraclass correlation coefficient; CI, confidence interval.

### MME and osteophytes in elderly subjects

Using the PDFS MRI and PPDFS MRI data, we analyzed the knee joints of all 1191 subjects in our cohort. As shown in Supplemental Figure 2, MME was determined using PDFS MRI, and osteophyte width was evaluated using PPDFS MRI. MME was observed in almost all subjects (98.7%; 1175 of 1191 subjects), and the mean ± standard deviation (SD) of MME width was 3.90 ± 2.06 mm (Table 2). The prevalence rates of medial femoral and tibial osteophytes (cartilage and bone parts of osteophytes) were 99.1% (1180 of 1191 subjects) and 99.3% (1183 of 1191 subjects), respectively (Table 2). The full-length width of the medial femoral osteophyte was 3.51 ± 1.98 mm, which was calculated by summing the cartilage-part width (1.39 ± 1.05 mm) and the bone-part width (2.13 ± 1.78 mm) of the medial femoral osteophyte in each subject (Table 2). Similarly, the full-length width of the medial tibial osteophyte was 3.27 ± 1.50 mm, and the width of cartilage and bone parts of osteophyte was 1.44 ± 1.09 mm and 1.83 ± 1.26 mm, respectively (Table 2).

**Table 2:** MME and osteophyte prevalence and width of the medial compartment of the knee joint of the subjects.

			Prevalence: number (%)	Width (mm): mean (SD)	Range (mm)
MME			1175 (98.7)	3.90 (2.06)	0–12.21
Osteophyte	Femur	Cartilage part	946 (79.4)	1.39 (1.05)	0–6.08
		Bone part	1055 (88.6)	2.13 (1.78)	0–10.48
		Cartilage and bone parts	1180 (99.1)	3.51 (1.98)	0–12.44
	Tibia	Cartilage part	921 (77.3)	1.44 (1.09)	0–6.14
		Bone part	1050 (88.2)	1.83 (1.26)	0–8.94
		Cartilage and bone parts	1183 (99.3)	3.27 (1.50)	0–10.18

*MME* medial meniscus extrusion.

### MME is most strongly associated with medial tibial osteophyte width among MRI-detected OA structural changes

We scored the OA structural changes observed by PDFS MRI according to Whole-Organ Magnetic Resonance Imaging Score (WORMS) and examined the associations between OA changes and MME (Table 3). All MRI-detected OA structural alterations, including those of cartilage, bone marrow lesions (BMLs), subchondral bone attrition (SBA), subchondral bone cysts (SBCs), osteophytes, meniscus, and the WORMS total score, were associated with MME. Furthermore, the bone-part and full-length widths of osteophytes in the femur were also correlated with MME (r = 0.410 [95% confidence interval [CI] 0.362–0.456] and r = 0.481 [95% CI 0.436–0.524], *p* < 0.001, respectively). Similarly, the bone-part and full-length width of osteophytes in the tibia were also correlated with MME (r = 0.473 [95% CI 0.428–0.516] and r = 0.809 [95% CI 0.788–0.828], *p* < 0.001, respectively) (Table 3). A multiple regression analysis indicated that, among these MRI-detected OA structural changes, the full-length width of the medial tibial osteophyte was the most closely associated with MME (β = 0.713; *p* < 0.001) (Table 4). When the subjects were divided into three subgroups according to the radiographic severity of knee OA, associations between MME and the full-length width of the medial tibial osteophyte were also observed in each subgroup (K/L1: r = 0.711, *p* < 0.001; K/L2: r = 0.764, *p* < 0.001; K/L3 and 4: r = 0.820, *p* < 0.001). These data suggest strong conformity between the MME and medial tibial osteophyte width.

**Table 3:** Associations between MME and MRI-detected OA structural alterations in elderly subjects.

MRI-detected OA structural alterations	r (95% CI)	*P* value
WORMS score		
Medial compartment		
Cartilage	0.305 (0.252 to 0.356)	< 0.001
BML	0.279 (0.226 to 0.331)	< 0.001
SBA	− 0.117 (− 0.173 to − 0.061)	< 0.001
SBC	0.259 (0.205 to 0.311)	< 0.001
Osteophyte	0.457 (0.411 to 0.501)	< 0.001
Meniscus	0.315 (0.263 to 0.366)	< 0.001
Total	0.515 (0.472 to 0.556)	< 0.001
Osteophyte width		
Femur (medial)		
Bone part	0.410 (0.362 to 0.456)	< 0.001
Cartilage and bone parts	0.481 (0.436 to 0.524)	< 0.001
Tibia (medial)		
Bone part	0.473 (0.428 to 0.516)	< 0.001
Cartilage and bone parts	0.809 (0.788 to 0.828)	< 0.001

*MME* medial meniscus extrusion, *CI* confidence interval, *WORMS* whole organ magnetic resonance imaging score, *BML* subchondral bone marrow abnormality, *SBA* subchondral bone attrition, *SBC* subchondral bone cyst.

**Table 4:** MRI-detected OA structural alterations and their associations with MME.

Factor	Univariable β	*P* value	Multivariable β	*P* value
WORMS score				
Medial compartment				
Cartilage	0.326	< 0.001	0.023	0.172
BML	0.545	< 0.001	0.000	0.986
SBA	− 0.245	< 0.001	− 0.019	0.206
SBC	0.612	< 0.001	0.009	0.588
Osteophyte	0.292	< 0.001	0.086	< 0.001
Meniscus	0.370	< 0.001	0.092	< 0.001
Osteophyte width				
Medial side of femur				
Bone part	0.677	< 0.001	0.033	0.269
Cartilage and bone parts	0.643	< 0.001	0.081	0.006
Medial side of tibia				
Bone part	0.961	< 0.001	− 0.036	0.088
Cartilage and bone parts	1.161	< 0.001	0.713	< 0.001

*MME* medial meniscus extrusion, *WORMS* whole organ magnetic resonance imaging score, *BML* subchondral bone marrow abnormality, *SBA* subchondral bone attrition, *SBC* subchondral bone cyst.

### Association of MME with the full-length width of medial tibial osteophytes

Next, we examined the conformity between the MME and the medial tibial osteophyte width. When the bone part of the osteophyte was evaluated, the ICC between the bone part width of the medial tibial osteophyte and the MME was 0.304 (95% CI − 0.079 to 0.576) (Fig. 1A). However, the ICC improved to 0.758 (95% CI 0.578–0.846) when the full-length width of osteophytes was evaluated (Fig. 1B). When the subjects were divided into three groups according to the difference between MME and the full-length width of medial tibial osteophytes, most of the subjects (96.7%; 1152 of 1191 subjects) showed an MME equal to or longer than the full-length width of osteophytes. Furthermore, 66.6% of subjects (793 of 1191 subjects) showed an MME equal to the full-length width of osteophytes ([MME]—[medial tibial osteophyte width] <  ± 1 mm), and 30.1% of subjects (359 of 1191 subjects) showed an MME longer than the full-length width of osteophytes ([MME]—[medial tibial osteophyte width] > 1 mm). Only a few subjects (3.3%; 39 of 1191 subjects) showed an MME shorter than the full-length width of osteophytes ([MME]—[medial tibial osteophyte width] < − 1 mm).

## Discussion

In the present study, we have demonstrated that MME and medial tibial osteophytes are constantly observed in elderly individuals who complain of no or weak knee joint symptoms, such as pain, and that the degree of MME is consistent with the full-length width of medial tibial osteophytes. Our data in the current study have confirmed the previous finding that radiographic knee OA is commonly observed in elderly people, showing a prevalence of knee OA with K/L grade ≥ 2 in more than 80% of women ≥ 80 years old^4^, and further provided, to the best of our knowledge, the first evidence that > 98% of elderly individuals exhibit MME and medial tibial osteophytes, which have a direct correlation with each other.

MME is a risk factor for knee OA incidence and progression^19,39,40^. In addition, we recently reported that among the MRI-detected structural changes of the OA knee joint, MME is a crucial factor for reduced walking speed^6^, which is one of the determinants of the remaining life span for elderly populations^41^. Since meniscus tear and/or meniscus degeneration causes elongation of the meniscus^42^, MME readily develops when knee joints suffer from these meniscal disorders^42^. However, MME is known to commonly occur without meniscus tear or destruction not only in early-stage knee OA but also in established knee OA patients^21,23–27^, suggesting a different mechanism for MME development from the meniscus problem. Although no data were available on the prevalence of MME in large-scale population-based cohort studies, our study using conventional PDFS MRI provides the first evidence that MME develops in almost all elderly individuals.

Osteophytes were originally thought to be a secondary process for repair after damage of the articular cartilage in knee OA^43^, but histological studies on experimental knee OA models demonstrated that osteophyte formation can be seen within two to three days after OA induction at a stage prior to cartilage destruction, indicating a fast process independent of cartilage repair^28^. Osteophytes play a role in joint stabilization and can be a source of pain and function loss^28^. In addition, accumulating evidence has revealed that osteophytes are a risk factor for the incidence and progression of knee OA^44–48^. Osteophytes are formed in the periosteum at the bone-cartilage junction of the joint following the process of the endochondral ossification and are composed of cartilage and bone parts. Thus, the diagnosis of osteophytes using radiographic and conventional PDFS MRI methods is disadvantageous because radiography is unable to detect cartilage tissue, and PDFS MRI is challenging in this regard, resulting in an underestimation of the incidence and size of osteophytes. To overcome this problem, T2 mapping MRI is known to be useful for the evaluation of osteophytes^21^, since this method can detect cartilage and its degeneration, which is reflected by the water content changes and disarrangement of type II collagen in the tissue. However, because of its relatively time-consuming nature, T2 mapping MRI is demanding for application in large-scale population-based cohort studies. Therefore, in the present study, we developed and applied the PPDFS MRI method to evaluate osteophytes. The PPDFS images were comparable to T2 mapping, showing a higher contrast resolution than T2 mapping images, and are applicable for the evaluation of osteophytes in cohort studies.

**Figure 2:**
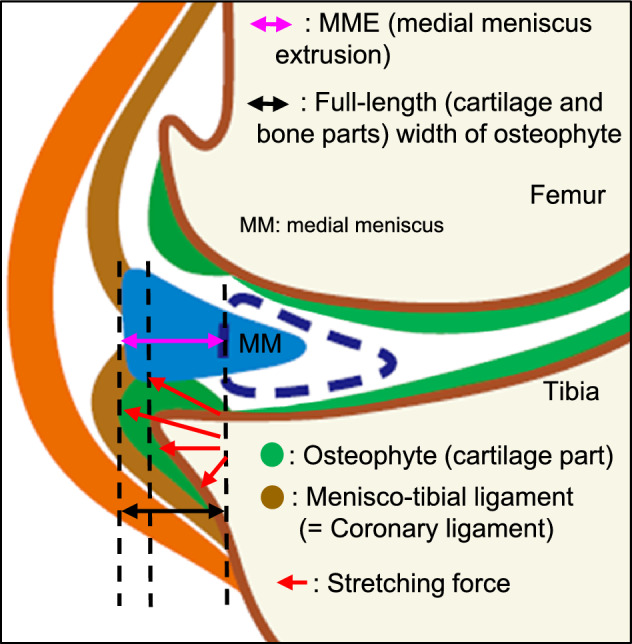
A schematic illustration showing the hypothesis on medial meniscus extrusion (MME) induced by osteophyte formed at the bone-cartilage junction of the medial tibia. Osteophytes are formed in the periosteum at the bone-cartilage junction following endochondral ossification and are thus composed of cartilage and bone parts. The most important anatomical characteristic is that the medial meniscus is tightly attached to the medial tibial plateau by the menisco-tibial ligament (coronary ligament). Therefore, the mechanical stretching force generated during joint movement because of the medial tibial osteophyte may contribute to the displacement of the medial meniscus by pressing the menisco-tibial ligament, leading to MME.

One of the important findings in the present study is that, among the OA structural changes, MME was highly consistent with the full-length width of medial tibial osteophytes determined by PPDFS MRI. In our previous study on early-stage knee OA patients, we found a similar correlation between MME and the width of medial tibial osteophytes measured by T2 mapping MRI and hypothesized that osteophytes may contribute to medial displacement of the meniscus, leading to MME^21^. As illustrated in Fig. 2, the anatomy of the knee joint indicates that the medial meniscus is tightly attached to the medial tibial plateau by the meniscotibial ligament, i.e. the coronary ligament^34,49^. Thus, once osteophytes are formed in the periosteum at the bone-cartilage junction of the medial tibia, it is plausible to speculate that the local mechanical stretching force occurring during joint movement may displace the medial meniscus to induce MME (Fig. 2). Although our previous study on early-stage knee OA analyzed only 50 cases, in the present study, we demonstrated that MME was equal to or longer than the full-length width of the osteophyte in 96.7% of subjects (1152 of 1191 subjects), and only a few cases (3.3%, 39 of 1191 subjects) showed an MME shorter than the osteophyte width. Since the introduction of MRI for the diagnosis of knee OA, growing evidence obtained from studies using MRI has demonstrated that osteophytes are formed at very early stages of knee OA^12,21^, suggesting that cartilage degradation and damage occur due to the load concentration onto the articular cartilage uncovered by the meniscus, leading to OA progression^39^. Taken together, one could speculate that osteophytes may contribute to the development of MME, which promotes meniscus injury and cartilage damage, resulting in OA progression^12,21^. However, further research is required to provide direct evidence supporting this hypothesis.

Although the present study demonstrated an extremely high incidence of MME and medial tibial osteophytes, both of which are directly correlated, evidence that osteophytes cause MME was not obtained because it was a cross-sectional study. However, since the PPDFS MRI method can be used to monitor changes in the osteophyte and meniscus by re-analyzing the ordinary MRI images already taken in these elderly patients, early-stage knee OA patients, or patients with anterior cruciate ligament injury, longitudinal studies on these subjects or patients, which are now underway in our group, would provide us with data on the relationship. After elucidating the causal relationship between osteophytes and MME, studies on the molecular mechanism of osteophyte formation and the search for their regulators^30,31,50–54^ might become important to develop disease-modifying therapies that can inhibit the progression of knee OA and/or prevent OA development.

Several limitations associated with the present study warrant mention. First, our cohort included only subjects living in an urban area in Japan. Therefore, the data obtained in the present study may not be applicable to elderly people living in other areas, such as mountainous or coastal areas, and the results cannot be generalized. Second, we developed the PPDFS MRI method by pseudo-colorization of PDFS images obtained from patients with symptomatic knee OA, but not from normal subjects. Therefore, whether or not this method is sufficient to evaluate subtle changes in the knee joints, including osteophytes, in healthy elderly individuals cannot be guaranteed. However, we have shown that the MRI views obtained by PPDFS MRI are almost equivalent to those obtained by T2 mapping MRI, and the widths of the cartilage and bone parts of osteophytes detected by PPDFS MRI were histologically confirmed. Because the method does not take much time for an analysis, it is suitable to evaluate numerous image samples using the ImageJ software program^55^. The accuracy of this method should be further assessed by analyzing the knee joints of human subjects, including cadavers. Third, the method of evaluating MRI-detected OA changes in the knee joint used in this study was limited. In particular, because the same observers measured both MME and osteophyte width, there is a risk of confirming our own hypothesis. This is one of the biases in the present study. In addition, while the accuracy of the PPDFS MRI method was evaluated using 3.0-Tesla MRI by measuring the osteophyte width of OA patients who received UKA, we measured the osteophyte width of the subjects in our cohort using 0.3-Tesla MRI. Therefore, we cannot exclude the possibility that the different MRI systems used for the analyses may have introduced a certain bias in the present study.

In conclusion, in elderly populations, MME and medial tibial osteophytes are frequently observed, and the degree of MME is consistent with the full-length width of the medial tibial osteophytes.

## Methods

### Subjects of the cohort study

We organized the BHS, a prospective cohort study over 10 years to identify risk factors for needing long-term care^35–38^, and recruited elderly subjects between 65 and 84 years old living in Bunkyo-ku, an urban area in Tokyo, Japan. A total of 1630 subjects participated from November 2015 to September 2018. The exclusion criteria of this cohort were elderly individuals who underwent pacemaker or defibrillator implantation and/or had diabetes requiring insulin therapy.

The study protocol was approved by the ethics committee of Juntendo University in November 2015 (Nos. 2015078, 2016138, 2016131, and 2017121). This study was conducted in accordance with the principles of the Declaration of Helsinki. All participants provided their written informed consent at orientation meetings. Participants were informed that they had the right to withdraw from the trial at any time. The collected data were coded with nonidentifying numbers and stored securely in password-protected files. Accessibility to files was limited to the principal investigators.

### The radiographic evaluation of knee OA

The radiographic OA severity was evaluated according to the Kellgren-Lawrence (K/L) classification^56^ of the weight-bearing anteroposterior radiographs of the femorotibial joint for both knees using the bilateral standing extended view and the weight-bearing posteroanterior radiographs of the femorotibial joint with the knee flexed^57^. The femorotibial angle (FTA) was evaluated based on weight-bearing anteroposterior radiographs of the lower limbs.

### Development of the PPDFS MRI method for evaluation of osteophytes

We previously reported the usefulness of T2 mapping MRI for the analysis of osteophytes, since T2 mapping MRI could detect the cartilage part as well as the bone part of osteophytes, so the full-length width (width of cartilage and bone parts) of osteophytes measured by T2 mapping MRI corresponded well to the width obtained by histology^21^. However, T2 mapping MRI is not commonly available in clinical situations and is still challenging for a large cohort study because it requires a longer scan time and requires longer post-imaging processing than conventional PDFS MRI. To overcome these limitations, we developed a new method, i.e. PPDFS MRI method^32^. For this method, MRI views of the knee joints obtained by conventional PDFS MRI were pseudo-colored by selecting “Royal” as the color, which allowed the cartilage to be represented by an intermediate color using the ImageJ software program^55^.

The accuracy of the PPDFS MRI method in measuring the full-length width of osteophytes was examined using 10 samples from 10 knee OA patients who underwent both PDFS MRI and T2 mapping MRI analyses before the operation and then underwent UKA (Supplemental Table 1). To compare the findings of MRI and histology of osteophytes in the knee joints, the knee joints were analyzed by a 3.0-Tesla MRI system (Siemens Magnetom Verio; Siemens Healthcare, Erlangen, Germany) using the following conditions: coronal T2FS spin-echo images, repetitive time [TR] / echo time [TE] = 2000/25 ms; slice thickness = 3.5 mm and in-plane resolution 0.36 × 0.36 mm; and sagittal T2FS spin-echo images, [TR] / [TE] = 2100/22 ms; slice thickness = 3.5 mm and in-plane resolution 0.36 × 0.36 mm. PPDFS MRI views were prepared by pseudo-coloring PDFS MRI using the ImageJ software program, as described previously^32^. T2 mapping MRI views were obtained as described in our previous study^21^ using the following conditions: coronal T2 mapping images, [TR] / [TE] = 1000/13.8, 27.6, 41.4, 55.2, and 69.0 ms; slice thickness, 3 mm; and in-plane resolution, 0.42 × 0.42 mm. The surgically removed tibial tissue was fixed in 10% buffered formalin. After decalcification, the osteophytes were cut vertically in the center from the outer border to the bottom, and serial paraffin sections were stained with hematoxylin and eosin and Safranin O. The width of the osteophytes was determined by measuring the cartilage and bone parts using the cellSens software program (Olympus, Tokyo, Japan).

The study protocol complied with the principles outlined in the Declaration of Helsinki and was approved by the Ethical Committee Review Board of Juntendo University (No. 15-074). Written informed consent was obtained from the patients with knee OA who underwent UKA.

### MRI settings in the cohort study

The knee joints of the subjects of the BHS were analyzed using a 0.3-Tesla MRI system (Hitachi AIRIS Vento; Hitachi Medical Corporation, Tokyo, Japan) with a knee coil. Coronal PDFS and PPDFS spin-echo sequences (TR / TE = 1500/21.2 ms; in-plane resolution = 0.39 × 0.39 mm^2^; section thickness = 3 mm) were used for MME and osteophyte measurement. MRI data were evaluated in the medial femorotibial joint, and OA morphological changes were scored according to the Whole Organ Magnetic Resonance Imaging Score (WORMS)^58^. Each region of a compartment received its own score, which was added together^21^. Tears in the medial meniscus were observed in 42% (499/1145) of the subjects, while the remaining 58% (692/1145) showed no damage in the medial meniscus (Kaneko, H., et al. manuscript in preparation).

### The evaluation of MME and osteophytes

Single slices of PPDFS MRI images presenting the largest width of the tibial plateau (greatest area of the medial spine) were selected for measurements of the MME and osteophyte width^59^. Observers who were blinded to the characteristics of the subjects measured MME and osteophyte width from the end of the normal tibial plateau to the outermost edge of the body of the meniscus and osteophyte end, respectively, using the ImageJ software program. The cartilage and bone partsof the osteophytes were measured separately.

### Reproducibility of measurements

One observer (TA) assessed all MME and osteophyte widths twice, and the second observer (AA) assessed 154 randomly selected knees. Intra-observer reliability (intra-class correlation coefficient) was 0.94 (95% confidence interval [CI] 0.94–0.95) for MME and 0.92 (95% CI 0.91–0.92) for osteophytes, and inter-observer reliability (inter-class correlation coefficient) was 0.97 (95% CI 0.96–0.98) for MME and 0.96 (95% CI 0.95–0.97) for osteophytes.

### Statistical analyses

The associations between MME and other MRI-detected OA structural alterations were analyzed using Spearman's rank correlation and multiple regression analyses. The consistency between MME and medial tibial osteophyte width was also analyzed using the ICC with a two-way random model and absolute agreement type. Statistical significance was set at *p* values < 0.05. All analyses were performed using the SPSS 27 software program (SPSS Institute, Chicago, IL, USA).

### Ethics approval and consent to participate

The study protocol was approved by the ethics committee of Juntendo University in November 2015 (Nos. 2015078, 2016138, 2016131, and 2017121). This study was conducted in accordance with the principles of the Declaration of Helsinki. All participants provided their written informed consent at orientation meetings. Participants were informed that they had the right to withdraw from the trial at any time. The collected data were coded with nonidentifying numbers and stored securely in password-protected files. Accessibility to files is limited to the principal investigators.

### Supplementary Information

Supplementary Information 1.

Supplementary Information 2.

## Data Availability

This study is a longitudinal study, and the data are not publicly available for use by the general public. The datasets generated and analyzed during the current study are available from the corresponding author upon reasonable request.
