# Supplementary material for: Medial meniscus extrusion is invariably observed and consistent with tibial osteophyte width in elderly populations: The Bunkyo Health Study
Source: Sci Rep. 2023 Dec 20;13:22805. doi: 10.1038/s41598-023-49868-7 (PMC10739745; doi:10.1038/s41598-023-49868-7)
Supplement: Supplementary file 1 — Supplementary Information 1. [file 41598_2023_49868_MOESM1_ESM.docx]

**Supplemental Table 1.** Characteristics of the patients with knee OA who underwent UKA

| N | 10 |
| --- | --- |
| Age (y) (SD, Range) | 74.2 (6.03, 63 - 83) |
| Gender (Male / Female) | 1 / 9 |
| BMI (kg/m^2^) (SD, Range) | 23.8 (2.2, 20.8 - 26.9) |
| Radiographic OA severity | K/L2: 2  K/L3: 8  K/L4: 1 |

K/L, Kellgren-Lawrence grade; UKA, uni-compartmental knee arthroplasty.

**Supplemental Table 2.** Medial tibial osteophyte width of the patients with knee OA who underwent UKA

|  |  | Histology | PDFS MRI | PPDFS MRI | T2 mapping MRI |
| --- | --- | --- | --- | --- | --- |
| Cartilage part | Mean (SD) (mm) | 1.44 (0.75) | 1.57 (0.62) | 1.56 (0.69) | 1.43 (0.68) |
|  | Median | 1.23 | 1.53 | 1.34 | 1.29 |
|  | Q1 - Q3 | 0.77 - 2.41 | 1.09 – 1.87 | 1.04 - 2.13 | 0.83 - 2.12 |
|  | Range | 0.53 - 2.47 | 0.77 – 2.86 | 0.66 - 2.80 | 0.50 - 2.44 |
| Cartilage and bone parts | Mean (SD) (mm) | 3.88 (1.67) | 4.41 (1.86) | 3.96 (1.55) | 4.03 (1.55) |
|  | Median | 3.37 | 4.08 | 3.75 | 3.85 |
|  | Q1 - Q3 | 2.46 - 5.25 | 2.92 - 5.89 | 2.71 - 4.92 | 2.46 - 5.25 |
|  | Range | 1.87 - 7.11 | 2.14 - 8.13 | 2.10 - 6.90 | 2.14 - 6.54 |

PPDFS, pseudo-colored proton density-weighted fat-suppression; UKA, uni-compartmental knee arthroplasty.

**Supplemental Table 3.** Comparison of the medial tibial osteophyte width of the patients with knee OA who underwent UKA

| ICC (95% CI) | Histology - PDFS | Histology - PPDFS | Histology - T2 mapping | PDFS - PPDFS | PDFS - T2 mapping | PPDFS - T2 mapping |
| --- | --- | --- | --- | --- | --- | --- |
| Cartilage part | 0.62 (0.05 - 0.89) | 0.94 (0.77 - 0.98) | 0.97 (0.88 - 0.99) | 0.71 (0.22 - 0.92) | 0.53  (-0.08 - 0.86) | 0.88 (0.62 - 0.97) |
| Cartilage and bone parts | 0.79 (0.38 - 0.94) | 0.97 (0.87 - 0.99) | 0.96 (0.87 - 0.99) | 0.84 (0.50 - 0.96) | 0.87 (0.58 - 0.97) | 0.97 (0.88 - 0.99) |

UKA, uni-compartmental knee arthroplasty; ICC, intraclass correlation coefficient; CI, confidence interval; PDFS, proton density-weighted fat-suppression; PPDFS, pseudo-colored proton density-weighted fat-suppression.
