# Supplementary material for: Medial meniscus extrusion is invariably observed and consistent with tibial osteophyte width in elderly populations: The Bunkyo Health Study
Source: Sci Rep. 2023 Dec 20;13:22805. doi: 10.1038/s41598-023-49868-7 (PMC10739745; doi:10.1038/s41598-023-49868-7)
Supplement: Supplementary file 2 — Supplementary Information 2. [file 41598_2023_49868_MOESM2_ESM.pptx]

## Slide 1
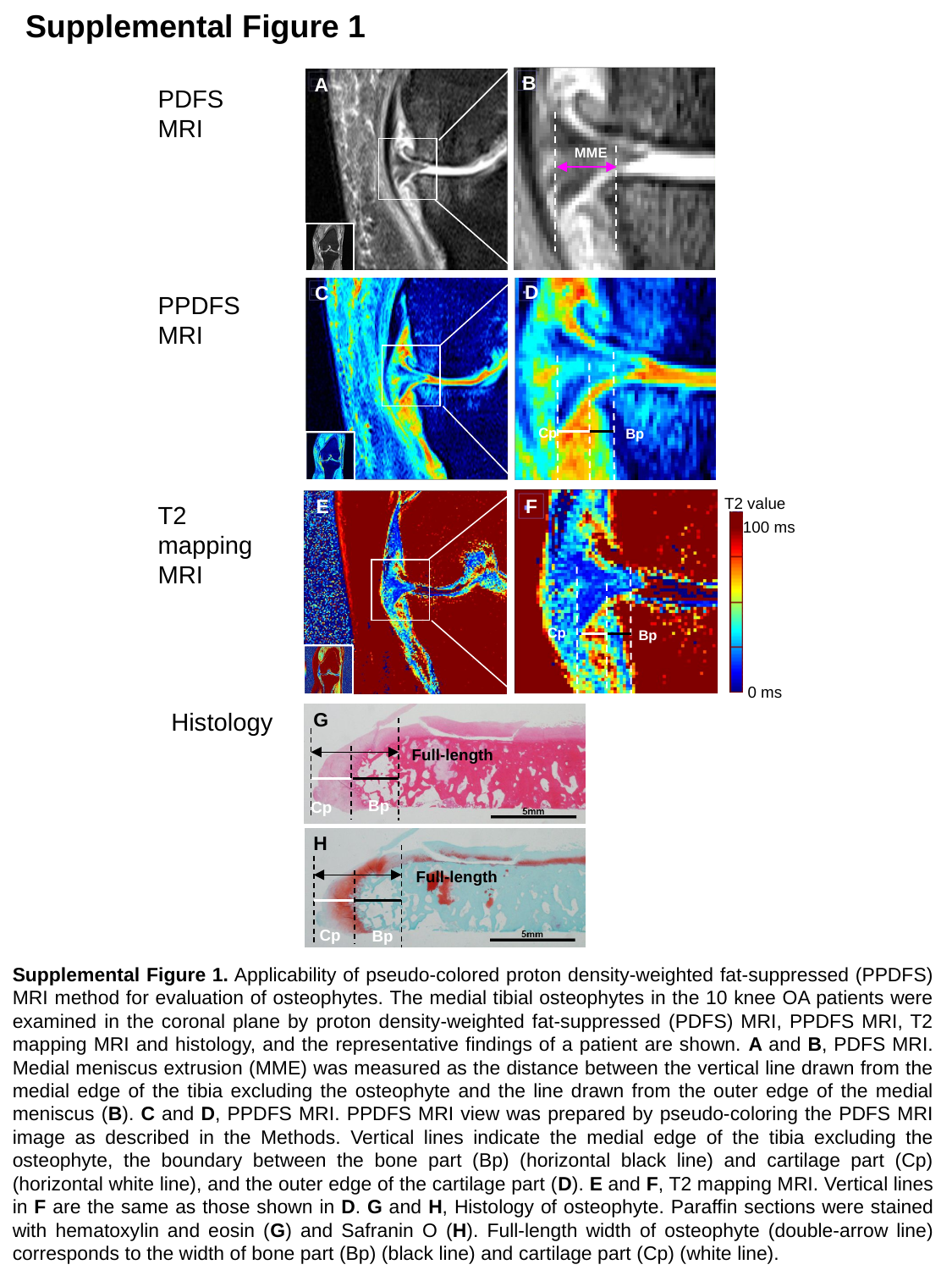

Supplemental Figure 1
B
A
PDFS MRI
MME
MME
D
C
PPDFS MRI
Cp
Bp
F
T2 value
100 ms
0 ms
E
F
T2 mapping MRI
Bp
Cp
Bp
Histology
G
Full-length
Bp
Cp
H
Full-length
Cp
Bp
Supplemental Figure 1. Applicability of pseudo-colored proton density-weighted fat-suppressed (PPDFS) MRI method for evaluation of osteophytes. The medial tibial osteophytes in the 10 knee OA patients were examined in the coronal plane by proton density-weighted fat-suppressed (PDFS) MRI, PPDFS MRI, T2 mapping MRI and histology, and the representative findings of a patient are shown. A and B, PDFS MRI. Medial meniscus extrusion (MME) was measured as the distance between the vertical line drawn from the medial edge of the tibia excluding the osteophyte and the line drawn from the outer edge of the medial meniscus (B). C and D, PPDFS MRI. PPDFS MRI view was prepared by pseudo-coloring the PDFS MRI image as described in the Methods. Vertical lines indicate the medial edge of the tibia excluding the osteophyte, the boundary between the bone part (Bp) (horizontal black line) and cartilage part (Cp) (horizontal white line), and the outer edge of the cartilage part (D). E and F, T2 mapping MRI. Vertical lines in F are the same as those shown in D. G and H, Histology of osteophyte. Paraffin sections were stained with hematoxylin and eosin (G) and Safranin O (H). Full-length width of osteophyte (double-arrow line) corresponds to the width of bone part (Bp) (black line) and cartilage part (Cp) (white line).

## Slide 2
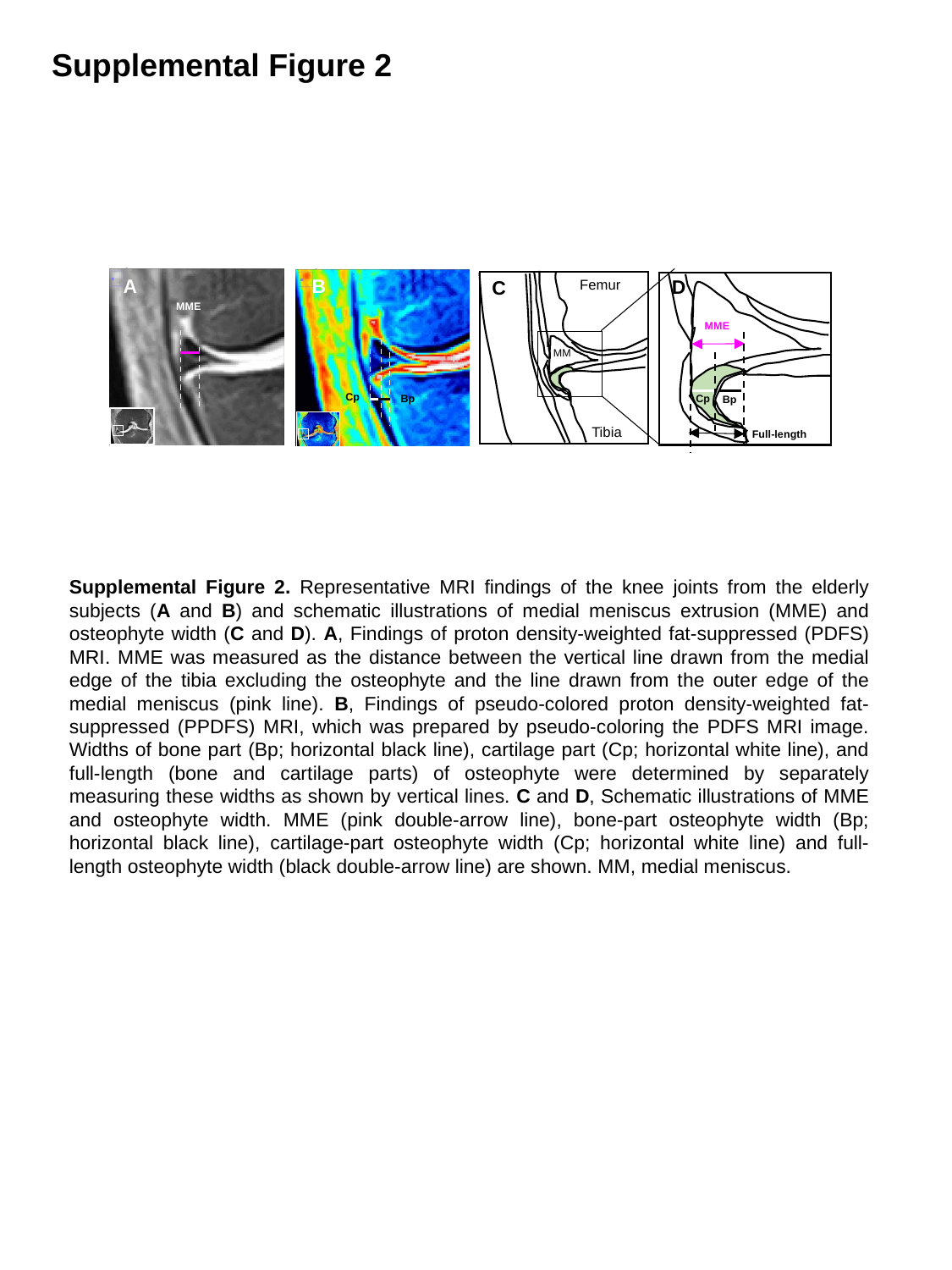

Supplemental Figure 2
A
B
D
C
Femur
MME
MME
MM
Cp
Bp
Cp
Bp
Tibia
Full-length
Supplemental Figure 2. Representative MRI findings of the knee joints from the elderly subjects (A and B) and schematic illustrations of medial meniscus extrusion (MME) and osteophyte width (C and D). A, Findings of proton density-weighted fat-suppressed (PDFS) MRI. MME was measured as the distance between the vertical line drawn from the medial edge of the tibia excluding the osteophyte and the line drawn from the outer edge of the medial meniscus (pink line). B, Findings of pseudo-colored proton density-weighted fat-suppressed (PPDFS) MRI, which was prepared by pseudo-coloring the PDFS MRI image. Widths of bone part (Bp; horizontal black line), cartilage part (Cp; horizontal white line), and full-length (bone and cartilage parts) of osteophyte were determined by separately measuring these widths as shown by vertical lines. C and D, Schematic illustrations of MME and osteophyte width. MME (pink double-arrow line), bone-part osteophyte width (Bp; horizontal black line), cartilage-part osteophyte width (Cp; horizontal white line) and full-length osteophyte width (black double-arrow line) are shown. MM, medial meniscus.
